# The joint association of diabetes status and NT-ProBNP with adverse cardiac outcomes in patients with non-ST-segment elevation acute coronary syndrome: a prospective cohort study

**DOI:** 10.1186/s12933-023-01771-1

**Published:** 2023-03-04

**Authors:** Man Wang, Wen Su, Hui Chen, Hongwei Li

**Affiliations:** 1 Department of Cardiology, Cardiovascular Center, Beijing Friendship Hospital, Capital Medical University, No.95, Yongan Road, Xicheng District, Beijing, 100050 People’s Republic of China; 2 Beijing Key Laboratory of Metabolic Disorder Related Cardiovascular Disease, Beijing, China

**Keywords:** Diabetes status, NT-proBNP, Non-ST-segment elevation acute coronary syndrome, Joint association

## Abstract

**Aims:**

To examine the joint association of diabetes status and N-terminal pro-B-type natriuretic peptide (NT-proBNP) with subsequent risk of major adverse cardio-cerebral events (MACCEs) and all-cause mortality in patients with non-ST-segment elevation acute coronary syndrome (NSTE-ACS).

**Methods:**

A total of 7956 NSTE-ACS patients recruited from the Cardiovascular Center Beijing Friendship Hospital Database Bank were included in this cohort study. Patients were divided into nine groups according to diabetes status (normoglycemia, prediabetes, diabetes) and NT-proBNP tertiles (< 92 pg/ml, 92–335 pg/ml, ≥ 336 pg/ml). Multivariable Cox proportional hazards models were used to estimate the individual and joint association of diabetes status and NT-proBNP with the risk of MACCEs and all-cause mortality.

**Results:**

During 20,257.9 person-years of follow-up, 1070 MACCEs were documented. In the fully adjusted model, diabetes and a higher level of NT-proBNP were independently associated with MACCEs risk (HR 1.42, 95% CI: 1.20–1.68; HR 1.72, 95% CI: 1.40–2.11) and all-cause mortality (HR 1.37, 95% CI: 1.05–1.78; HR 2.80, 95% CI: 1.89–4.17). Compared with patients with normoglycemia and NT-proBNP < 92 pg/ml, the strongest numerical adjusted hazards for MACCEs and all-cause mortality were observed in patients with diabetes and NT-proBNP ≥ 336 pg/ml (HR 2.67, 95% CI: 1.83–3.89; HR 2.98, 95% CI: 1.48–6.00). The association between MACCEs and all-cause mortality with various combinations of NT-proBNP level, HbA1c, and fasting plasma glucose was studied.

**Conclusions:**

Diabetes status and elevated NT-proBNP were independently and jointly associated with MACCEs and all-cause mortality in patients with NSTE-ACS.

**Supplementary Information:**

The online version contains supplementary material available at 10.1186/s12933-023-01771-1.

## Introduction

Diabetes has become a major health concern in China, the prevalence of which increased from 9.7% in 2007 to 11.2% in 2017 among adults [1]. Besides being a risk factor for the development of coronary artery disease (CAD), diabetes is also strongly associated with an increased risk for subsequent adverse cardiovascular events in patients with acute coronary syndrome (ACS) [2–4]. In addition, diabetes is related to a high risk of multivessel coronary artery disease and has a rising prevalence in individuals with a non-ST-segment elevation ACS (NSTE-ACS) [5, 6]. Revascularization is one of the major treatments for NSTE-ACS, but the optimal revascularization strategy (percutaneous coronary intervention [PCI] vs. coronary artery bypass grafting [CABG]) remains controversial due to the lack of randomized comparison [7]. Thus, early and precise risk stratification, as one of the factors in the decision-making process, is essential for the prognosis of patients with NSTE-ACS.

Recently, N-terminal pro-B-type natriuretic peptide (NT-proBNP) has received attention as a biomarker of cardiac reserve and hemodynamic stress [8]. NT-proBNP has been found a strong and independent predictor of subsequent adverse cardiovascular events in the spectrum of ACS patients [9–11]. The current guideline from the European Society of Cardiology (ESC) has newly recommended that measuring NT-proBNP plasma concentrations should be considered to gain prognostic information for the risk assessment and management of patients with NSTE-ACS [12]. In addition, a few studies have indicated that NT-proBNP may help the cardiologist to select either PCI or CABG as the revascularization strategy in patients with left main CAD or three-vessel CAD [13–15]. However, fewer studies evaluated the prognostic value of NT-proBNP in patients with NSTE-ACS combined with glycemic status. One previous study has confirmed a similar extent of association between NT-proBNP and increased risk of mortality across the spectrum of diabetes status in a community population [16]. However, less is known about the joint association of diabetes status and NT-proBNP with mortality in the acute scenario of NSTE-ACS.

Thus, the present study aims to extend previous observations and comprehensively evaluate the joint association of diabetes status and NT-proBNP with subsequent risk of cardiovascular events in a large Chinses cohort of patients with NSTE-ACS. We hypothesized that patients with both diabetes and elevated NT-proBNP would be associated with an increased risk of adverse cardiac outcomes.

## Methods

### Study population

**Fig. 1:**
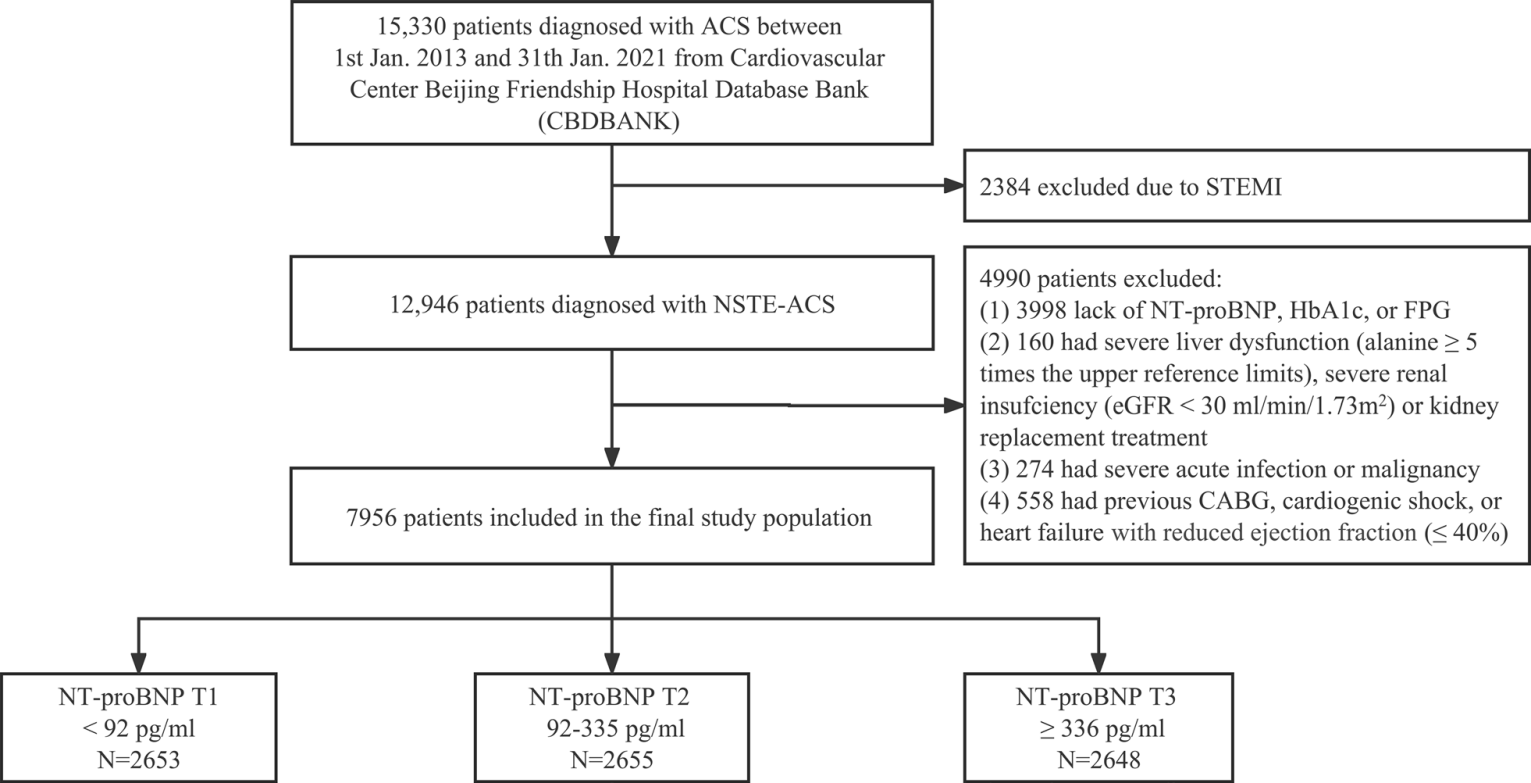
Study flow chart

The CBDBANK (Cardiovascular Center Beijing Friendship Hospital Database Bank) is a prospective cohort study of 15,330 consecutive patients diagnosed with ACS from January 2013 to January 2021. A total of 12,946 patients were diagnosed with NSTE-ACS (including non-ST-segment elevation myocardial infarction [NSTEMI] and unstable angina [UA]) based on the guideline [12]. Of the 12,946 patients, 4990 were excluded according to the exclusion criteria, which were (1) lack of NT-proBNP, fasting plasma glucose (FPG), or glycosylated hemoglobin (HbA1c), (2) severe liver dysfunction (alanine ≥ 5 times the upper reference limits), severe renal insufficiency (estimated glomerular filtration rate [eGFR] < 30 ml/min/1.73m^2^), or kidney replacement treatment, (3) severe acute infection or malignancy, and (4) previous CABG, cardiogenic shock or heart failure with reduced ejection fraction (left ventricular ejection fraction [LVEF] ≤ 40%). Cardiogenic shock was defined as systolic blood pressure (SBP) < 90 mmHg for ≥ 30 min or catecholamines to maintain SBP > 90 mmHg, and clinical pulmonary congestion and impaired end-organ perfusion (altered mental status, cold/clammy skin and extremities, urine output < 30 ml/h, or lactate > 2.0 mmol/l), or a class IV rating according to the Killip classification [17, 18]. Overall, 7956 patients were included in this study (Fig. 1).

The study was approved by the Ethics Committee of Beijing Friendship Hospital, Capital Medical University, and was following the Declaration of Helsinki.

### Measurements of NT-ProBNP

Blood samples were drawn from patients during hospitalization and immediately measured using the Chemiluminescent Enzyme Immuno Assay (PATHFAST™ Immunoanalyzer, PHC Europe B.V.). The coefficients of variation for the assays range from 4.6% to 5.4%. The lower and upper limits of detection are 15 pg/ml and 30,000 pg/ml, respectively. This study used the peak values of NT-proBNP for further analyses as a continuous variable and as a category variable based on the NT-proBNP tertiles (T1 < 92 pg/ml, T2 = 92–335 pg/ml, T3 ≥ 336 pg/ml) following previous studies [19, 20].

### Glycemic measures

Overnight fasting venous blood samples were collected and immediately transferred to the central laboratory (Beijing Friendship Hospital) for HbA1c and FPG testing using standard laboratory methods. The classifications of normoglycemia, prediabetes, and diabetes were based primarily on the American Diabetes Association (ADA) criteria [21]: diabetes was defined as previously diagnosed with diabetes, the use of anti-diabetic medications, FPG ≥ 7.0 mmol/l, or HbA1c ≥ 6.5%; prediabetes was as FPG 5.6–6.9 mmol/l or HbA1c 5.7–6.4%; and normoglycemia was as FPG < 5.6 mmol/l and HbA1c < 5.7%.

### Outcome

The primary endpoint was major adverse cardio-cerebral events (MACCEs), including all-cause death, non-fatal myocardial infarction, non-fatal stroke, and ischemia-induced revascularization within 5 years after discharge. The secondary outcome was all-cause mortality. Non-fatal stroke was defined as signs of neurological dysfunction caused by cerebral vascular obstruction or sudden rupture confirmed by computed tomography or magnetic resonance imaging. Any revascularization was defined as percutaneous intervention or bypass surgery of the target vessel or non-target vessels. Incident cardiovascular events during hospitalization were confirmed by medical record review. Clinical follow-up was performed by telephone interview or outpatient follow-up.

### Covariates

Baseline characteristics on demographic and clinical information, including age, sex, medical history, lifestyles (smoking status [none, ever, current], body mass index [BMI]), laboratory results, and in-hospital therapy were collected from hospital records. Medical history, including previous hypertension, dyslipidemia, myocardial infarction, stroke, and PCI, was obtained according to the self-reported history of diagnosis. Trained staff measured systolic and diastolic blood pressure, heart rate, as well as weight and height. BMI was calculated as weight in kilograms divided by height in meters squared. Overnight fasting blood samples were obtained and examined for biomarkers including hemoglobin, serum creatinine, high-sensitivity C-reactive protein (hs-CRP), triglycerides (TG), total cholesterol (TC), low-density lipoprotein cholesterol (LDL-C), and high-density lipoprotein cholesterol (HDL-C). Elevated hs-CRP was defined as ≥ 3 mg/l according to the Centers for Disease Control and Prevention and the American Heart Association [22]. The eGFR was calculated using the MDRD (The Modification of Diet in Renal Disease) formula: eGFR (ml/min/1.73 m^2^) = 175 × (Scr)^−1.154^ × (Age)^−0.203^ × (0.742 if female) × (1.212 if African American) [23]. Furthermore, according to National Kidney Foundation criteria, patients were classified into two eGFR groups: eGFR ≥ 60 ml/min /1.73m^2^ and eGFR < 60 ml/min/1.73m^2^ [24]. Echocardiograms were performed by expert cardiologists or ultrasound specialists. The LVEF was measured using the Simpsons method, and patients were classified into LVEF categories (41–45%, 46–50%, 51–55%, 56–60%, 61–65%, 66–70%, or > 70%) [25]. The coronary angiography and PCI operation were implemented according to relevant guidelines by experienced cardiologists. Standard medications during hospitalization were obtained directly from the medical records, including antiplatelet therapy (aspirin, or clopidogrel/ticagrelor), β-blocker, angiotensin-converting enzyme inhibitor (ACEI) or angiotensin receptor blocker (ARB), and statins.

### Statistical analysis

Continuous normally distributed variables were summarized as mean ± SD, while medians (P_25_-P_75_) were reported for non-normally distributed variables. Categorical variables were reported as frequency and percentage. Study participants were categorized by NT-proBNP tertiles, and baseline characteristics were compared using one-way ANOVA, Kruskal–Wallis H test, or Pearson’s χ^2^ test as appropriate.

We first calculated incidence rates of MACCEs and all-cause mortality (per 1000 person-years) by categories of diabetes status and NT-proBNP. We also graphically illustrated the cumulative incidence of MACCEs and all-cause mortality by categories of NT-proBNP according to diabetes status using the Kaplan–Meier method, and differences were compared by the Log-rank test.

Using multivariable Cox proportional hazards models and after stratification into subgroups of (1) diabetes status (normoglycemia, prediabetes, diabetes); (2) HbA1c categories (< 5.7%, 5.7–6.4%, ≥ 6.5%); or (3) FPG categories (< 5.6 mmol/l, 5.6–6.9 mmol/l, ≥ 7.0 mmol/l), we estimated the hazard ratios (HRs) and 95% confidence intervals (CIs) for the associations of NT-proBNP (modeled as a categorical or continuous variable) with MACCEs and all-cause mortality. To explore the joint association of diabetes status and NT-proBNP, we created a new variable by combining diabetes status and NT-proBNP, which had nine categories representing nine (3 × 3) combinations of diabetes status (normoglycemia, prediabetes, and diabetes) and NT-proBNP level (low, medium, and high). A similar method was conducted to create two new variables representing the combinations of NT-proBNP level, HbA1c, and FPG categories. Multivariable models were adjusted for age, sex, BMI, NSTE-ACS status, previous hypertension, previous dyslipidemia, previous myocardial infarction, SBP, heart rate, LVEF, eGFR, hs-CRP, LDL-C, smoking status, and in-hospital treatments (PCI, antiplatelet therapy, β-blocker, ACEI or ARB, and statins). Separate Cox models were conducted according to the outcomes and glycemic measures for analysis of NT-proBNP. The reference groups were selected: (1) NT-proBNP < 92 pg/ml and normoglycemia; (2) NT-proBNP < 92 pg/ml and HbA1c < 5.7%; (3) NT-proBNP < 92 pg/ml and FPG < 5.6 mmol/L. The *P* values for trends were calculated based on the results of the Wald χ^2^ test on the linearity hypothesis of ordered NT-proBNP or glycemic categories. The *P* values for interactions between categories of NT-proBNP and diabetes status, HbA1c, or FPG categories for the association of outcomes were also estimated using the Wald χ^2^ test by adding an interaction term (i.e., NT-proBNP × glycemic categories) in the multivariable models. We also used restricted cubic spline analyses to examine the relationship between NT-proBNP as a continuous variable and the risk of outcomes according to each glycemic category.

Because NT-proBNP level is strongly associated with sex, age, and BMI [26–28], analyses stratified by sex (male, female), age group (< 60, ≥ 60 years), and BMI category (< 25, ≥ 25 kg/m^2^) were also conducted. We also evaluated the risk of MACCEs and all-cause mortality by cross-categories of NT-proBNP tertiles and diabetes status—including further categorization according to glycemic control (HbA1c < 7% vs HbA1c ≥ 7%), with NT-proBNP < 92 pg/ml and no diabetes as the reference.

Analysis was performed using Stata software, version 17.0 (StataCorp LP, College Station, TX, USA), and R software, version 4.1.2 (R Foundation for Statistical Computing). A 2-sided *P*-value < 0.05 was considered to be statistically significant.

## Results

### Baseline characteristics

Of the 7956 patients in the current study, the mean ± SD age of the study population was 65.4 ± 10.5 years, and 62.7% were male. The median (IQR) levels of NT-proBNP of the whole cohort were 165.0 (65.8, 527.5). Table 1 presents the baseline characteristics according to NT-proBNP tertiles. Participants with higher NT-proBNP were more likely to be older and NSTEMI; have diabetes, previous hypertension, previous myocardial infarction, previous stroke, and previous PCI; have higher levels of FPG, HbA1c and hs-CRP; have lower levels of BMI, diastolic blood pressure, LVEF, hemoglobin, triglyceride, and eGFR; and have a higher rate of receiving clopidogrel or ticagrelor, β-Blocker, ACEI/ARB and PCI during hospitalization.

**Table 1:** Baseline and clinical characteristics by NT-proBNP categories

	Total	T1 (< 92 pg/ml)	T2 (92–335 pg/ml)	T3 (≥ 336 pg/ml)	*P* value
Number	7956	2653	2655	2648	
Clinical characteristics					
Age, year	65.4 ± 10.5	60.8 ± 8.6	65.9 ± 9.7	69.6 ± 11.0	< 0.001
Male, n (%)	4986 (62.7)	1885 (71.1)	1547 (58.3)	1554 (58.7)	< 0.001
BMI, kg/m^2^	25.9 ± 3.6	26.2 ± 3.4	26.0 ± 3.7	25.5 ± 3.7	< 0.001
Heart rate, bpm	71.4 ± 12.2	71.3 ± 10.6	69.6 ± 10.9	73.3 ± 14.5	< 0.001
SBP, mmHg	132.9 ± 18.1	130.1 ± 15.5	133.7 ± 17.5	135.1 ± 20.7	< 0.001
DBP, mmHg	75.6 ± 11.6	77.1 ± 11.1	75.3 ± 11.4	74.4 ± 12.0	< 0.001
Diabetes status, n (%)					
Normoglycemia	1848 (23.2)	687 (25.9)	637 (24.0)	524 (19.8)	< 0.001
Prediabetes	2360 (29.7)	822 (31.0)	777 (29.3)	761 (28.7)	
Diabetes	3748 (47.1)	1144 (43.1)	1241 (46.7)	1363 (51.5)	
Previous hypertension, n (%)	5736 (72.1)	1786 (67.3)	1955 (73.6)	1995 (75.3)	< 0.001
Previous dyslipidemia, n (%)	3861 (48.5)	1357 (51.1)	1335 (50.3)	1169 (44.1)	< 0.001
Previous MI, n (%)	630 (7.9)	114 (4.3)	201 (7.6)	315 (11.9)	< 0.001
Previous stroke, n (%)	1462 (18.4)	350 (13.2)	501 (18.9)	611 (23.1)	< 0.001
Previous PCI, n (%)	1200 (15.1)	318 (12.0)	429 (16.2)	453 (17.1)	< 0.001
Current smoker, n (%)	2606 (32.8)	997 (37.6)	791 (29.8)	818 (30.9)	< 0.001
LVEF, %	65.6 ± 6.8	67.6 ± 4.8	66.6 ± 5.9	62.5 ± 8.1	< 0.001
NSTE-ACS status, n (%)					
UA	6183 (77.7)	2532 (95.4)	2271 (85.5)	1380 (52.1)	< 0.001
NSTEMI	1773 (22.3)	121 (4.6)	384 (14.5)	1268 (47.9)	
Laboratory examinations					
FPG, mmol/L	6.1 ± 2.2	6.0 ± 1.9	6.1 ± 2.1	6.3 ± 2.4	< 0.001
HbA1c, %	6.5 ± 1.4	6.4 ± 1.3	6.5 ± 1.4	6.6 ± 1.4	< 0.001
Hemoglobin, g/l	133.9 ± 17.9	140.1 ± 15.1	133.4 ± 16.2	128.2 ± 20.1	< 0.001
eGFR, ml/min/1.73m^2^	112.7 ± 30.5	124.0 ± 27.0	113.7 ± 28.5	100.4 ± 31.2	< 0.001
eGFR < 60 ml/min/1.73m^2^, n (%)	324 (4.1)	16 (0.6)	65 (2.4)	243 (9.2)	< 0.001
hs-CRP, mg/l	1.7 (0.7, 5.3)	1.1 (0.5, 2.8)	1.4 (0.6, 3.9)	3.4 (1.2, 12.3)	< 0.001
hs-CRP ≥ 3 mg/l, n (%)	2696 (33.9)	568 (21.4)	785 (29.6)	1343 (50.7)	< 0.001
Peak value of NT-proBNP, pg/ml	165.0 (65.8, 527.5)	46.9 (29.7, 65.8)	165.0 (122.0, 227.0)	983.5 (528.0, 2380.0)	< 0.001
Triglyceride, mmol/l	1.36 (0.99, 1.93)	1.46 (1.05, 2.04)	1.37 (1.00, 1.95)	1.28 (0.94, 1.81)	< 0.001
Total cholesterol, mmol/l	4.23 ± 1.05	4.21 ± 1.03	4.23 ± 1.04	4.26 ± 1.09	0.320
LDL-C, mmol/l	2.39 ± 0.75	2.36 ± 0.72	2.37 ± 0.75	2.42 ± 0.78	0.007
HDL-C, mmol/l	1.09 ± 0.27	1.08 ± 0.25	1.10 ± 0.27	1.07 ± 0.27	< 0.001
In-hospital treatment, n (%)					
Aspirin	7125 (89.6)	2416 (91.1)	2418 (91.1)	2291 (86.5)	< 0.001
Clopidogrel/Ticagrelor	4420 (55.6)	1212 (45.7)	1447 (54.5)	1761 (66.5)	< 0.001
β-Blocker	5240 (65.9)	1648 (62.1)	1705 (64.2)	1887 (71.3)	< 0.001
ACEI/ARB	4222 (53.1)	1155 (43.5)	1404 (52.9)	1663 (62.8)	< 0.001
Statins	7064 (88.8)	2387 (90.0)	2377 (89.5)	2300 (86.9)	< 0.001
PCI	3957 (49.7)	1145 (43.2)	1339 (50.4)	1473 (55.6)	< 0.001

Values are mean ± SD, n (%), or median (interquartile interval)

ACEI, angiotensin-converting enzyme inhibitor; ARB, angiotensin receptor blocker; BMI, body mass index; DBP, diastolic blood pressure; eGFR, estimated glomerular filtration rate; FPG, fasting plasma glucose; HbA1c, glycosylated hemoglobin; HDL-C, high-density lipoprotein cholesterol; hs-CRP, high sensitivity C-reactive protein; LDL-C, low-density lipoprotein cholesterol; LVEF, left ventricular ejection fraction; MI, myocardial infarction; NSTEMI, non-ST-segment elevation myocardial infarction; NSTE-ACS, non-ST-segment elevation acute coronary syndrome; NT-proBNP, N-terminal pro-B-type natriuretic peptide; PCI, percutaneous coronary intervention; SBP, systolic blood pressure; UA, unstable angina

During 20,257.9 person-years of follow-up, 13.5% of the study population experienced a first MACCEs (n = 1070; 52.8 per 1000 person-years). This included 461 incidents of all-cause death (21.3 per 1000 person-years), 253 incidents of non-fatal myocardial infarction (12.0 per 1000 person-years), 111 incidents of non-fatal stroke (5.2 per 1000 person-years), and 434 incidents of ischemia-induced revascularization (21.0 per 1000 person-years). Approximately, 17.6% of MACCEs occurred in patients with normoglycemia (n = 1848) and 16.9% in patients with NT-proBNP < 92 pg/ml (n = 2653). In contrast, 56.0% of those with baseline NT-proBNP ≥ 336 pg/ml (n = 2648) experienced a MACCEs during follow-up.

### Independent association of NT-ProBNP, diabetes status, and outcomes

In the whole cohort, patients with diabetes were associated with a higher risk of MACCEs and all-cause mortality, with adjusted HRs of 1.42 (95% CI: 1.20–1.68) and 1.37 (95% CI: 1.05–1.78), respectively (Table 2). In addition, a higher level of NT-proBNP was significantly related to a higher risk of MACCEs and all-cause mortality. The adjusted HRs were 1.00 (reference), 1.24 (95% CI: 1.02–1.50) and 1.72 (95% CI: 1.40–2.11) for MACCEs, and 1.00 (reference), 1.47 (95% CI: 0.98–2.21) and 2.80 (95% CI: 1.89–4.17) for all-cause mortality across the NT-proBNP tertiles, respectively (Table 2). Besides, patients with higher levels of NT-proBNP also had an elevated risk of cardiovascular mortality, non-fatal MI, and revascularization (Additional file 1: Figure S1).

**Table 2:** Number of events, incidence rates, and adjusted HRs for cardiovascular outcomes across the spectrum of diabetes status or NT-proBNP categories

	Events/population	Incidence rate per 1000 person‑years (95% CI)	Adjusted HR (95% CI) *
MACCEs			
Diabetes status			
Normoglycemia	188/1848	38.7 (33.6–44.7)	Ref.
Prediabetes	289/2360	48.7 (43.4–54.7)	1.15 (0.95–1.39)
Diabetes	593/3748	62.6 (57.8–67.9)	**1.42 (1.20–1.68)**
NT-proBNP tertiles			
T1 < 92 pg/ml	181/2653	27.8 (24.0–32.1)	Ref.
T2 92–335 pg/ml	290/2655	41.8 (37.2–46.9)	**1.24 (1.02–1.50)**
T3 ≥ 336 pg/ml	599/2648	88.2 (81.4–95.5)	**1.72 (1.40–2.11)**
All-cause mortality			
Diabetes status			
Normoglycemia	79/1848	15.5 (12.4–19.3)	Ref.
Prediabetes	119/2360	18.9 (15.8–22.6)	1.00 (0.75–1.34)
Diabetes	263/3748	25.7 (22.8–29.0)	**1.37 (1.05–1.78)**
NT-proBNP tertiles			
T1 < 92 pg/ml	34/2653	5.0 (3.6–7.0)	Ref.
T2 92–335 pg/ml	87/2655	11.8 (9.6–14.6)	1.47 (0.98–2.21)
T3 ≥ 336 pg/ml	340/2648	45.7 (41.1–50.8)	**2.80 (1.89–4.17)**

*Covariates included in the model were age, sex, BMI, NSTE-ACS status, previous hypertension, previous dyslipidemia, previous myocardial infarction, systolic blood pressure, heart rate, LVEF, eGFR, hs-CRP, LDL-C, smoking status, and in-hospital treatments (PCI, antiplatelet therapy, β-blocker, ACEI or ARB, and statins); Statistically significant estimates in bold

Abbreviations see Table 1

### NT-ProBNP, diabetes status, and outcomes

**Fig. 2:**
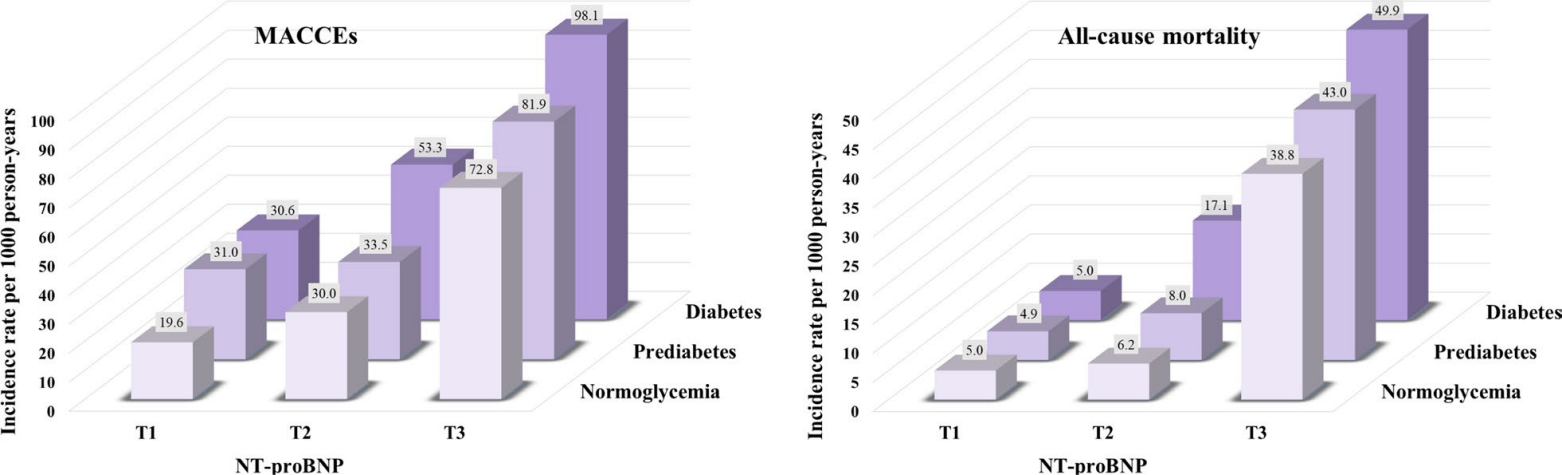
Incidence rate per 1000 person-years of MACCEs and mortality in different subgroups of diabetes status and NT-proBNP categories

**Fig. 3:**
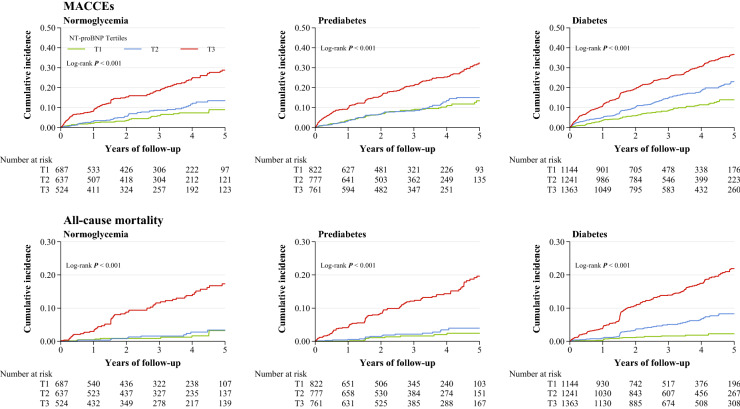
Kaplan–Meier estimated event rates of MACCEs and all-cause mortality by NT-proBNP tertiles across different diabetes statuses

**Fig. 4:**
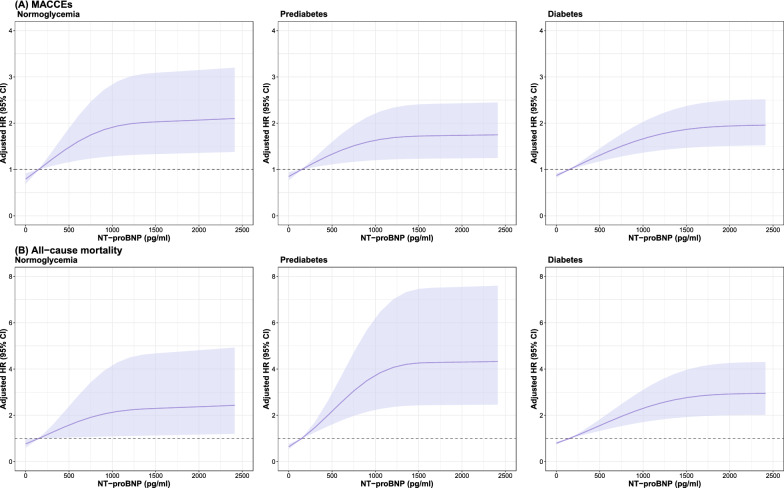
Restricted cubic spline analysis for association of the NT-proBNP and risk of MACCEs and all-cause mortality across different diabetes statuses. Adjusted model included age, sex, BMI, NSTE-ACS status, previous hypertension, previous dyslipidemia, previous myocardial infarction, systolic blood pressure, heart rate, LVEF, eGFR, hs-CRP, LDL-C, smoking status, and in-hospital treatments (PCI, antiplatelet therapy, β-blocker, ACEI or ARB, and statins)

Incidence rates of MACCEs and all-cause mortality (for 1000 person-years) by cross categories of NT-proBNP tertiles and diabetes status were shown in Fig. 2. Event rates for MACCEs and all-cause mortality were lowest in those with NT-proBNP < 92 pg/ml and normoglycemia. The cumulative incidence of first MACCEs and all-cause mortality was higher in patients with baseline NT-proBNP ≥ 336 pg/ml compared to those with NT-proBNP < 92 pg/ml across each diabetes category (Log-rank *P* < 0.001 for all; Fig. 3). Using restricted cubic spline analyses, we observed an approximately positive linear relationship between NT-proBNP on a continuous scale and MACCEs as well as all-cause mortality across each diabetes status, after adjustment for possible confounders (Fig. 4).

**Fig. 5:**
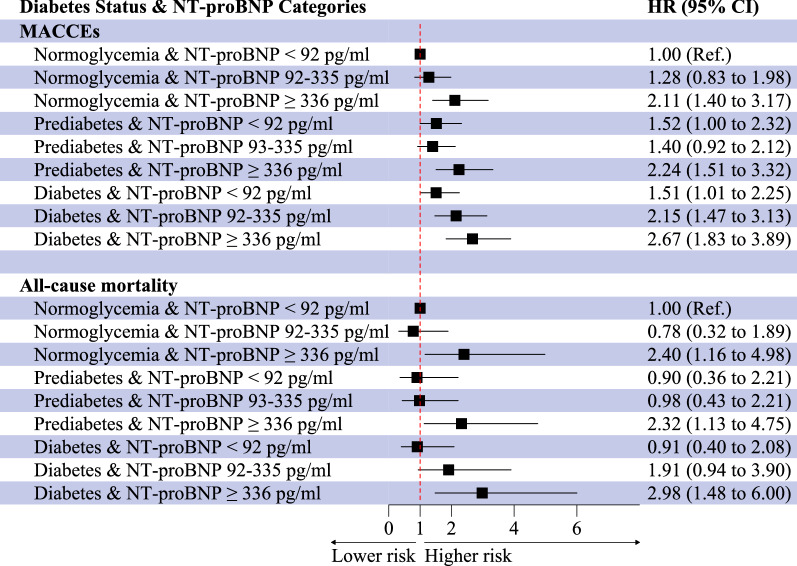
Forest plots of the adjusted hazard ratios of MACCEs and all-cause mortality across diabetes status and NT-proBNP categories. The HR was calculated using Cox proportional hazards model adjusted for age, sex, BMI, NSTE-ACS status, previous hypertension, previous dyslipidemia, previous myocardial infarction, systolic blood pressure, heart rate, LVEF, eGFR, hs-CRP, LDL-C, smoking status, and in-hospital treatments (PCI, antiplatelet therapy, β-blocker, ACEI or ARB, and statins)

Table 3 and Fig. 5 show the joint associations between diabetes status and NT-proBNP categories with incident MACCEs and all-cause mortality. After adjustment of potential confounders, increasing levels of NT-proBNP were associated with higher incidents of MACCEs and all-cause mortality across each diabetes status. Compared with patients with normoglycemia and a low NT-proBNP level, the strongest numerical hazards for MACCEs and all-cause mortality were seen in patients with diabetes and NT-proBNP ≥ 336 pg/ml (HR 2.67, 95% CI: 1.83–3.89; HR 2.98, 95% CI: 1.48–6.00; Table 3, Fig. 5). However, there was no significant interaction between diabetes status and NT-proBNP for risk of MACCEs and all-cause mortality (Additional file 1: Table S1). We also observed similar results in subgroup analyses stratified by sex, age, and BMI (Additional file 1: Table S2).

**Table 3:** Adjusted HRs of cardiovascular outcomes across the spectrum of diabetes status and NT-proBNP categories

Diabetes status	NT-proBNP						
	T1 (< 92 pg/ml)		T2 (92–335 pg/ml)		T3 (≥ 336 pg/ml)		*P* value for trend
	n/N	HR (95% CI) *	n/N	HR (95% CI) *	n/N	HR (95% CI) *	
MACCEs							
Normoglycemia	34/687	Ref.	51/637	1.28 (0.83–1.98)	103/524	**2.11 (1.40–3.17)**	0.001
Prediabetes	60/822	**1.52 (1.00–2.32)**	68/777	1.40 (0.92–2.12)	161/761	**2.24 (1.51–3.32)**	< 0.001
Diabetes	87/1144	**1.51 (1.01–2.25)**	171/1241	**2.15 (1.47–3.13)**	335/1363	**2.67 (1.83–3.89)**	< 0.001
*P* value for trend		0.702		0.004		< 0.001	
All-cause mortality							
Normoglycemia	9/687	Ref.	11/637	0.78 (0.32–1.89)	59/524	**2.40 (1.16–4.98)**	< 0.001
Prediabetes	10/822	0.90 (0.36–2.21)	17/777	0.98 (0.43–2.21)	92/761	**2.32 (1.13–4.75)**	< 0.001
Diabetes	15/1144	0.91 (0.40–2.08)	59/1241	1.91 (0.94–3.90)	189/1363	**2.98 (1.48–6.00)**	< 0.001
*P* value for trend		0.056		0.442		< 0.001	

^*^Estimates were adjusted for age, sex, BMI, NSTE-ACS status, previous hypertension, previous dyslipidemia, previous myocardial infarction, systolic blood pressure, heart rate, LVEF, eGFR, hs-CRP, LDL-C, smoking status, and in-hospital treatments (PCI, antiplatelet therapy, β-blocker, ACEI or ARB, and statins); Statistically significant estimates in bold

Abbreviations see Table 1

When further stratified by glycemic control, among patients with elevated NT-proBNP (≥ 336 pg/ml), the adjusted HRs of MACCEs were 2.62 (95% CI: 1.77–3.89) and 2.72 (95% CI: 1.84–4.02) for diabetes with HbA1c < 7% and HbA1c ≥ 7%, respectively. Similarly, the HRs of all-cause mortality were 2.95 (95% CI: 1.44–6.03) for those with diabetes and HbA1c < 7%, and 3.02 (95% CI: 1.48–6.16) for those with diabetes and HbA1c ≥ 7% (Additional file 1: Table S3). We observed no clear interaction between the two variables for MACCEs (*P* for interaction = 0.088) and all-cause mortality (*P* for interaction = 0.364).

### NT-ProBNP, HbA1c, and outcomes

Increased NT-proBNP categories (≥ 336 pg/ml vs. < 92 pg/ml) demonstrated consistently higher event rates and significantly increased hazards for MACCEs and all-cause mortality across HbA1c strata (Table 4). Compared with patients with NT-proBNP < 92 pg/ml and HbA1c < 5.7%, the adjusted HRs of MACCEs and all-cause mortality were 2.72 (95% CI: 1.89–3.91) and 2.92 (95% CI: 1.53–5.57) for those with NT-proBNP ≥ 336 pg/ml and HbA1c ≥ 6.5%, respectively. We observed no clear interaction between HbA1c and NT-proBNP for outcomes (Additional file 1: Table S1). HbA1c ≥ 6.5% (vs. HbA1c < 5.7%) was associated with an increased risk of incident MACCEs in each NT-proBNP category, whereas the association was not observed for all-cause mortality in patients with NT-proBNP < 92 pg/ml (HR 0.92; 95% CI: 0.40–2.09; Table 4).

**Table 4:** Adjusted HRs of cardiovascular outcomes across the spectrum of HbA1c and NT-proBNP categories

HbA1c category	NT-proBNP						
	T1 (< 92 pg/ml)		T2 (92–335 pg/ml)		T3 (≥ 336 pg/ml)		*P* value for trend
	n/N	HR (95% CI) *	n/N	HR (95% CI) *	n/N	HR (95% CI) *	
MACCEs							
< 5.7%	39/798	Ref.	58/719	1.28 (0.85–1.93)	130/641	**2.19 (1.50–3.20)**	< 0.001
5.7–6.4%	69/978	1.44 (0.97–2.14)	88/962	**1.46 (1.00–2.15)**	211/954	**2.41 (1.67–3.47)**	< 0.001
≥ 6.5%	73/877	**1.71 (1.16–2.53)**	144/974	**2.40 (1.67–3.46)**	258/1053	**2.72 (1.89–3.91)**	< 0.001
*P* value for trend		0.357		0.001		< 0.001	
All-cause mortality							
< 5.7%	11/798	Ref.	12/719	0.71 (0.31–1.62)	76/641	**2.42 (1.25–4.69)**	< 0.001
5.7–6.4%	11/978	0.75 (0.33–1.74)	24/962	0.99 (0.48–2.04)	118/954	**2.26 (1.18–4.34)**	< 0.001
≥ 6.5%	12/877	0.92 (0.40–2.09)	51/974	**2.07 (1.06–4.03)**	146/1053	**2.92 (1.53–5.57)**	< 0.001
*P* value for trend		0.048		0.333		< 0.001	

^*^Estimates were adjusted for age, sex, BMI, NSTE-ACS status, previous hypertension, previous dyslipidemia, previous myocardial infarction, systolic blood pressure, heart rate, LVEF, eGFR, hs-CRP, LDL-C, smoking status, and in-hospital treatments (PCI, antiplatelet therapy, β-blocker, ACEI or ARB, and statins); Statistically significant estimates in bold

Abbreviations see Table 1

### NT-ProBNP, FPG, and outcomes

Table 5 shows the joint associations of NT-proBNP and FPG categories with incident MACCEs and all-cause mortality, selecting patients with a low NT-proBNP and FPG level as the reference. Within each FPG category, a higher category of NT-proBNP was associated with significantly increased risk for incident MACCEs and all-cause mortality (Table 5). Patients with the highest NT-proBNP (≥ 336 pg/ml) and FPG (≥ 7.0 mmol/l) had the highest numerical hazards for MACCEs (HR 2.33; 95% CI: 1.74–3.12) and all-cause mortality (HR 3.27; 95% CI: 1.92–5.57) compared with patients with NT-proBNP < 92 pg/ml and FPG < 5.6 mmol/l (Table 5). There was a marginally significant interaction between FPG and NT-proBNP for MACCEs, but not for all-cause mortality (Additional file 1: Table S1).

**Table 5:** Adjusted HRs of cardiovascular outcomes across the spectrum of FPG and NT-proBNP categories

FPG category	NT-proBNP						
	T1 (< 92 pg/ml)		T2 (92–335 pg/ml)		T3 (≥ 336 pg/ml)		
	n/N	HR (95% CI) *	n/N	HR (95% CI) *	n/N	HR (95% CI) *	*P* value for trend
MACCEs							
< 5.6 mmol/l	86/1533	Ref.	128/1486	1.18 (0.89–1.56)	302/1365	**2.02 (1.55–2.65)**	< 0.001
5.6–6.9 mmol/l	52/630	1.40 (0.99–1.98)	78/635	**1.63 (1.19–2.24)**	126/623	**1.85 (1.36–2.50)**	< 0.001
≥ 7.0 mmol/l	43/490	1.37 (0.95–1.97)	84/534	**1.96 (1.44–2.68)**	171/660	**2.33 (1.74–3.12)**	< 0.001
* P* value for trend		0.932		0.013		< 0.001	
All-cause mortality							
< 5.6 mmol/l	19/1533	Ref.	36/1486	1.12 (0.64–1.97)	177/1365	**2.77 (1.66–4.61)**	< 0.001
5.6–6.9 mmol/l	8/630	0.96 (0.42–2.20)	17/635	1.23 (0.63–2.38)	73/623	**2.61 (1.52–4.50)**	< 0.001
≥ 7.0 mmol/l	7/490	0.98 (0.41–2.33)	34/534	**2.66 (1.49–4.72)**	90/660	**3.27 (1.92–5.57)**	< 0.001
* P* value for trend		0.062		0.341		0.002	

^*^Estimates were adjusted for age, sex, BMI, NSTE-ACS status, previous hypertension, previous dyslipidemia, previous myocardial infarction, systolic blood pressure, heart rate, LVEF, eGFR, hs-CRP, LDL-C, smoking status, and in-hospital treatments (PCI, antiplatelet therapy, β-blocker, ACEI or ARB, and statins); Statistically significant estimates in bold

Abbreviations see Table 1

## Discussion

To our knowledge, this is the first study that prospectively evaluated the joint association of diabetes status and NT-proBNP with the subsequent risk of MACCEs and all-cause mortality in patients with NSTE-ACS. Compared with individuals with normoglycemia and NT-proBNP < 92 pg/ml, individuals with NT-proBNP ≥ 336 pg/ml and diabetes, HbA1c ≥ 6.5%, or FPG ≥ 7.0 mmol/l, were at higher risk of first MACCEs and all-cause mortality. We did not observe a significant interaction between diabetes status and NT-proBNP for incident MACCEs and all-cause mortality.

The prognostic value of NT-proBNP for adverse cardiovascular outcomes is increasingly identified in patients with diabetes. One recent study of a community-based cohort of 5861 individuals provided compelling evidence that NT-proBNP alone was superior to conventional risk factors for the prediction of cardiovascular events [29]. In addition, Prausmüller et al. evaluated the predictive performance of NT-proBNP with the recently published ESC/European Association for the Study of Diabetes (EASD) risk stratification model and the Systemic Coronary Risk Evaluation (SCORE) in patients with type 2 diabetes [30]. In this study, compared to the ESC/EASD and SCORE risk model, NT-proBNP remained a robust predictor for predicting 10-year cardiovascular disease and all-cause mortality in individuals with type 2 diabetes [30]. One case-cohort study within the European Prospective Investigation Into Cancer and Nutrition (EPIC)-Potsdam cohort indicated that NT-proBNP was positively associated with diabetes-related microvascular and macrovascular complications, which could be useful in monitoring the risk of vascular complications [31]. In general, these studies highlighted the extremely high prognostic value of NT-proBNP to identify high-risk patients with diabetes. However, none of these studies analyzed the joint association of diabetes status and NT-proBNP in patients with existing cardiovascular diseases. Our study extended these findings to patients with NSTE-ACS and found that patients with both a higher glycemic category and NT-proBNP level were associated with worse outcomes.

The association of increased NT-proBNP levels with adverse outcomes was observed among numerous studies regarding the NSTE-ACS population [11, 32, 33]. It has been reported that plasma NT-proBNP would rise rapidly from the onset of myocardial ischemia due to acute left ventricular dysfunction [34]. The increased magnitude of NT-proBNP level is proportional to the severity of myocardial ischemia and subsequent left ventricular systolic and diastolic dysfunction [35]. In addition, prior studies also indicated that the prognostic value of NT-proBNP was on top of cardiac troponin [33]. Similar results were observed in the present study that NT-proBNP was independently associated with a higher risk of MACCEs and all-cause mortality in the whole cohort. Moreover, no significant interaction between diabetes status and NT-proBNP was observed, suggesting that NT-proBNP was also useful for further risk stratification within each diabetes status for patients with NSTE-ACS.

Remarkably, the latest ESC guideline has recommended using NT-proBNP to gain more prognostic information in patients with NSTE-ACS (Class of Recommendation: IIa) [12]. In addition, a recent consensus report of the ADA also suggested that an initial assessment of NT-proBNP could be used as a first-line screening tool [36]. The relationship between diabetes and NT-proBNP was described as a “partners in crime” relationship by one previous study [37]. Consistently, our study emphasized the joint effect of diabetes and NT-proBNP on the cardiovascular risk in patients with NSTE-ACS, with the highest hazards for MACCEs and all-cause mortality shown in patients with diabetes and NT-proBNP ≥ 336 pg/ml. Furthermore, among patients with diabetes and elevated NT-proBNP, higher HRs were observed in patients with unsatisfactory glycemic control (HbA1c ≥ 7%). These results highlighted the importance of glycemic control and the detection of NT-proBNP. We assumed that our findings could help improve the early identification of high-risk patients and lead to the application of the most appropriate treatments as soon as possible.

The potential underlying mechanisms of the joint effect of diabetes status and NT-proBNP remains unknown. Several longitudinal observational studies of population-based cohorts have shown that heart failure risk was enhanced two- to fivefold in patients with diabetes or prediabetes compared with those without [38, 39]. In the diabetes community, heart failure was widely recognized as one of the main complications, the frequency of which was second only to peripheral arterial disease [40]. Individuals with diabetes may develop “diabetic cardiomyopathy,” defined as left ventricular systolic or diastolic dysfunction in the absence of coronary artery disease and hypertension [41]. The mechanisms are complex and include several dysregulated pathways such as mitochondrial dysfunction, altered insulin signaling, oxidative stress, and increased formation of advanced glycation end products, all leading to functional and structural changes in the diabetic heart [41]. Therefore, cardiac dysfunction may be accelerated in the presence of diabetes in NSTE-ACS patients. Our results indicated that patients with baseline diabetes and NT-proBNP ≥ 336 pg/ml should be alert for further decline of cardiac function.

The strengths of this study included the large population, prospective design, long follow-up period, and the wide variety of adjustments of covariates. However, several limitations need to be addressed. Overnight fasting venous blood samples for FPG testing were obtained on the second day after admission and the diagnosis of diabetes may be overestimated due to stress hyperglycemia. Second, only a single measurement of NT-proBNP was used in the study, and potential bias due to measurement error should be considered. Thus, further longitudinal analyses should be performed to confirm these findings. Furthermore, details on the severity of myocardial ischemia or infarct size were failed to obtain in this study, thus residual or unmeasured confounders may exist.

## Conclusions

Diabetes status and higher levels of NT-proBNP were significantly associated with a higher risk of MACCEs and all-cause mortality in patients with NSTE-ACS. Detection of NT-proBNP would be useful to the prognostic evaluation and risk stratification, especially for patients with prediabetes and diabetes. Further randomized controlled trials are required to confirm whether intensification of treatment based on the joint association of diabetes status and NT-proBNP might improve the prognosis of patients with NSTE-ACS.

## Supplementary Information

**Additional file 1: Figure S1.** Kaplan-Meier curves for MACCEs, all-cause mortality, cardiovascular mortality, non-fatal MI, non-fatal stroke, and revascularization according to NT-proBNP categories. **Table S1.** P-value of the interaction of glycemic and NT-proBNP categories in the prediction of incident MACCEs and all-cause mortality. **Table S2.** Adjusted HRs of incident MACCE across the spectrum of diabetes status and NT-proBNP categories for different subgroups. **Table S3.** Adjusted HR of cardiovascular outcomes across the spectrum of glycemic control and NT-proBNP categories.

## Data Availability

The datasets used for the present analysis may be made available upon reasonable request by contacting the corresponding author.
