# Supplementary material for: The joint association of diabetes status and NT-ProBNP with adverse cardiac outcomes in patients with non-ST-segment elevation acute coronary syndrome: a prospective cohort study
Source: Cardiovasc Diabetol. 2023 Mar 4;22:46. doi: 10.1186/s12933-023-01771-1 (PMC9985841; doi:10.1186/s12933-023-01771-1)
Supplement: Supplementary file 1 — Additional file 1: Figure S1. Kaplan-Meier curves for MACCEs, all-cause mortality, cardiovascular mortality, non-fatal MI, non-fatal stroke, and revascularization according to NT-proBNP categories. Table S1. P-value of the interaction of glycemic and NT-proBNP categories in the prediction of incident MACCEs and all-cause mortality. Table S2. Adjusted HRs of incident MACCE across the spectrum of diabetes status and NT-proBNP categories for different subgroups. Table S3. Adjusted HR of cardiovascular outcomes across the spectrum of glycemic control and NT-proBNP categories. [file 12933_2023_1771_MOESM1_ESM.docx]

**Additional material**

**Additional Figure 1.** Kaplan-Meier curves for MACCEs, all-cause mortality, cardiovascular mortality, non-fatal MI, non-fatal stroke, and revascularization according to NT-proBNP categories


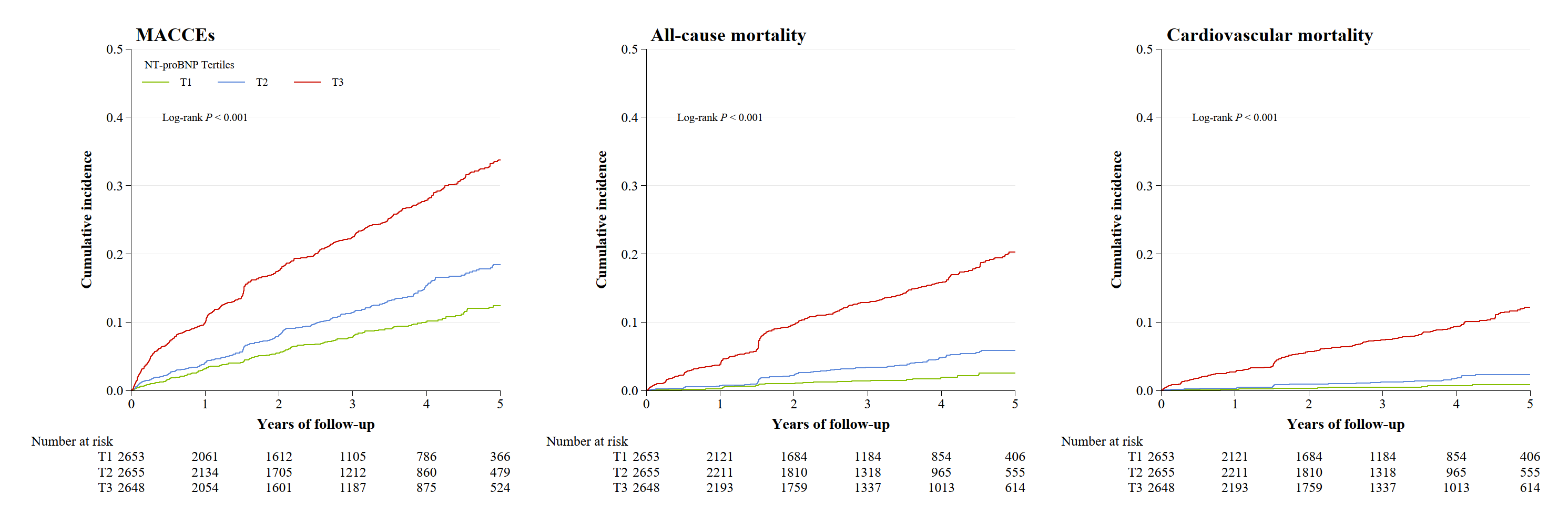


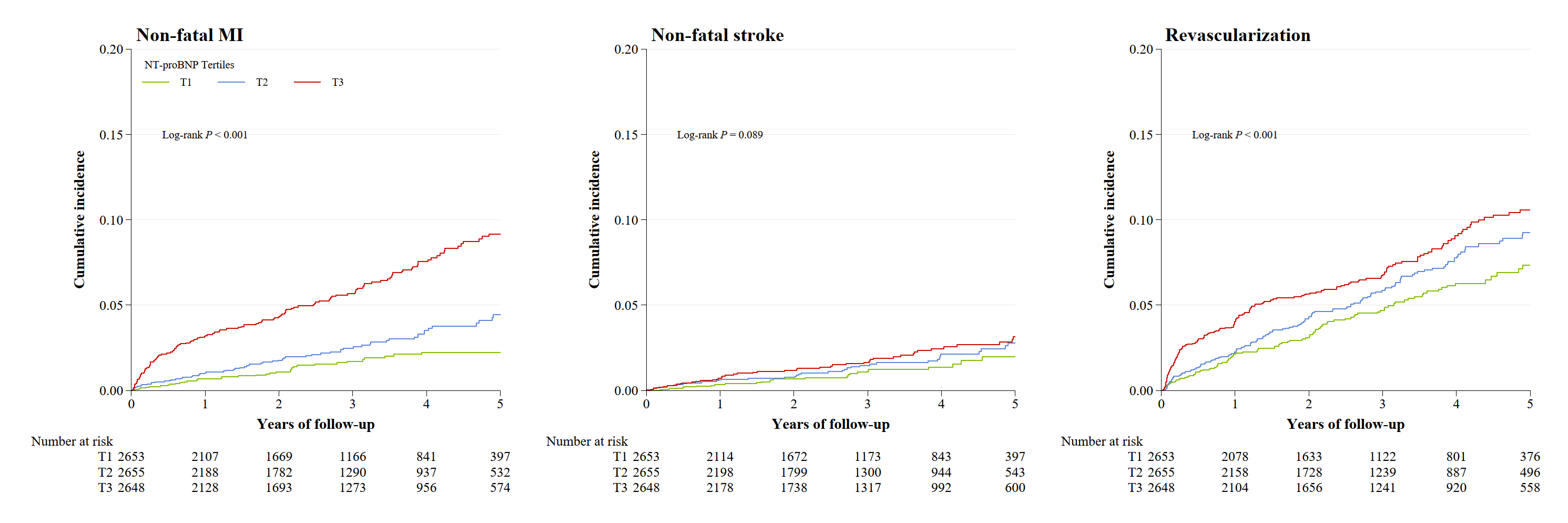


Abbreviations: MACCEs, major adverse cardio-cerebral events; MI, myocardial infarction; NT-proBNP, N-terminal pro-B-type natriuretic peptide.

| **Additional Table 1. *P*-value of the interaction of glycemic and NT-proBNP categories in the prediction of incident MACCEs and all-cause mortality** | | |
| --- | --- | --- |
| NT-proBNP categories × diabetes status | MACCEs | 0.1852 |
|  | All-cause mortality | 0.2250 |
| NT-proBNP risk categories × HbA1c categories | MACCEs | 0.0685 |
|  | All-cause mortality | 0.0780 |
| NT-proBNP risk categories × FPG categories | MACCEs | 0.0484 |
|  | All-cause mortality | 0.1184 |
| Abbreviations: FPG, fasting plasma glucose; HbA1c, glycosylated hemoglobin; MACCEs, major adverse cardio-cerebral events; NT-proBNP, N-terminal pro-B-type natriuretic peptide. | | |

| **Additional Table 2. Adjusted HRs of incident MACCE across the spectrum of diabetes status and NT-proBNP categories for different subgroups** | | | | | | | | |
| --- | --- | --- | --- | --- | --- | --- | --- | --- |
|  | **NT-proBNP** | | | | | | |  |
|  | **T1**  **< 92 pg/ml** | **T2**  **92-335 pg/ml** | **T3**  **≥ 336 pg/ml** | ***P* value for trend** | **T1**  **< 92 pg/ml** | **T2**  **92-335 pg/ml** | **T3**  **≥ 336 pg/ml** | ***P* value for trend** |
| **Diabetes status** | **HR (95% CI) *** | | |  | **HR (95% CI) *** | | |  |
|  | **Male (n = 4986)** | | |  | **Female (n = 2970)** | | |  |
| Normoglycemia | Ref. | 1.62 (0.99-2.66) | **2.11 (1.29-3.43)** | 0.116 | Ref. | 0.59 (0.22-1.53) | 1.91 (0.87-4.20) | < 0.001 |
| Prediabetes | **1.97 (1.23-3.16)** | 1.54 (0.94-2.52) | **2.47 (1.55-3.94)** | 0.008 | 0.55 (0.21-1.47) | 1.05 (0.47-2.37) | 1.70 (0.79-3.69) | < 0.001 |
| Diabetes | **1.81 (1.15-2.84)** | **2.54 (1.64-3.94)** | **2.64 (1.69-4.12)** | < 0.001 | 0.75 (0.32-1.77) | 1.40 (0.66-2.95) | **2.21 (1.05-4.66)** | < 0.001 |
| *P* value for trend | 0.183 | 0.003 | < 0.001 |  | 0.045 | 0.487 | < 0.001 |  |
|  | **Age < 60 y (n = 2288)** | | |  | **Age ≥ 60 y (n = 5668)** | | |  |
| Normoglycemia | Ref. | **2.12 (1.08-4.17)** | 1.32 (0.59-2.95) | 0.325 | Ref. | 1.17 (0.65-2.09) | **2.92 (1.74-4.89)** | < 0.001 |
| Prediabetes | 1.41 (0.74-2.70) | 1.55 (0.77-3.13) | 1.94 (0.95-3.97) | 0.125 | 1.65 (0.94-2.89) | 1.58 (0.92-2.71) | **3.03 (1.83-5.03)** | < 0.001 |
| Diabetes | 1.68 (0.93-3.02) | **1.91 (1.03-3.54)** | **3.13 (1.66-5.88)** | 0.002 | 1.42 (0.83-2.46) | **2.59 (1.58-4.25)** | **3.33 (2.03-5.44)** | < 0.001 |
| *P* value for trend | 0.323 | 0.133 | < 0.001 |  | 0.183 | 0.020 | < 0.001 |  |
|  | **BMI < 25 kg/m^2^ (n = 3398)** | | |  | **BMI ≥ 25 kg/m^2^ (n = 4558)** | | |  |
| Normoglycemia | Ref. | 1.37 (0.75-2.48) | **1.80 (1.01-3.19)** | 0.024 | Ref. | 1.15 (0.60-2.20) | **2.50 (1.40-4.47)** | 0.008 |
| Prediabetes | 1.32 (0.69-2.50) | 1.23 (0.67-2.28) | **2.29 (1.31-4.00)** | 0.002 | 1.66 (0.94-2.93) | 1.49 (0.84-2.64) | **2.10 (1.20-3.67)** | 0.004 |
| Diabetes | 1.34 (0.74-2.44) | **2.23 (1.30-3.84)** | **2.32 (1.35-3.99)** | < 0.001 | 1.60 (0.93-2.76) | **2.13 (1.26-3.59)** | **2.89 (1.71-4.90)** | < 0.001 |
| *P* value for trend | 0.757 | 0.031 | 0.001 |  | 0.491 | 0.034 | < 0.001 |  |
| *Estimates were adjusted for age, sex, BMI, NSTE-ACS status, previous hypertension, previous dyslipidemia, previous myocardial infarction, systolic blood pressure, heart rate, LVEF, eGFR, hs-CRP, LDL-C, smoking status, and in-hospital treatments (PCI, antiplatelet therapy, β-blocker, ACEI or ARB, and statins).  Abbreviations see Table 1. | | | | | | | | |

| **Additional Table 3. Adjusted HR of cardiovascular outcomes across the spectrum of glycemic control and NT-proBNP categories** | | | | |
| --- | --- | --- | --- | --- |
|  | **NT-proBNP** | | | |
|  | **HR (95% CI) *** | | | ***P* value for trend** |
| **Diabetes status** | T1  < 92pg/ml | T2  92-335 pg/ml | T3  ≥ 336 pg/ml |  |
| **MACCEs** |  | | |  |
| Normoglycemia | Ref. | 1.28 (0.83-1.98) | **2.12 (1.41-3.18)** | 0.001 |
| Prediabetes | **1.52 (1.00-2.32)** | 1.40 (0.92-2.12) | **2.24 (1.51-3.33)** | < 0.001 |
| Diabetes |  |  |  |  |
| HbA1c < 7.0% | 1.06 (0.64-1.75) | **1.90 (1.26-2.88)** | **2.62 (1.77-3.89)** | < 0.001 |
| HbA1c ≥ 7.0% | **1.90 (1.24-2.90)** | **2.39 (1.60-3.56)** | **2.72 (1.84-4.02)** | < 0.001 |
| *P* value for trend | 0.470 | 0.001 | < 0.001 |  |
| **All-cause mortality** |  |  |  |  |
| Normoglycemia | Ref. | 0.78 (0.32-1.89) | **2.41 (1.16-4.98)** | < 0.001 |
| Prediabetes | 0.90 (0.36-2.21) | 0.98 (0.43-2.21) | **2.32 (1.13-4.75)** | < 0.001 |
| Diabetes |  |  |  |  |
| HbA1c < 7.0% | 0.67 (0.22-2.01) | 1.73 (0.80-3.71) | **2.95 (1.44-6.03)** | < 0.001 |
| HbA1c ≥ 7.0% | 1.11 (0.45-2.73) | 2.10 (0.99-4.45) | **3.02 (1.48-6.16)** | < 0.001 |
| *P* value for trend | 0.072 | 0.386 | < 0.001 |  |
| *Estimates were adjusted for age, sex, BMI, NSTE-ACS status, previous hypertension, previous dyslipidemia, previous myocardial infarction, systolic blood pressure, heart rate, LVEF, eGFR, hs-CRP, LDL-C, smoking status, and in-hospital treatments (PCI, antiplatelet therapy, β-blocker, ACEI or ARB, and statins).  Abbreviations see Table 1. | | | | |
